# The SIK1-NR4A2 axis: a core driver of epilepsy identified by integrated machine learning and experimental validation

**DOI:** 10.3389/fcell.2026.1887268

**Published:** 2026-10-22

**Authors:** Xiuying Liu, Shouhuan Zheng, Kunling Yang, Lanfeng Sun, Lina Su, Shanmei Zhong, Huayue Liang, Xiaodan Luo

**Affiliations:** 1 Department of Neurology, The First People’s Hospital of Qinzhou, Qinzhou, Guangxi, China; 2 Department of Neurology, The First Affiliated Hospital of Guangxi Medical University, Nanning, Guangxi, China; 3 Department of Research, The First People’s Hospital of Qinzhou, Qinzhou, Guangxi, China

**Keywords:** epilepsy, LKB1 signaling pathway, machine learning, Nr4a2, SIK1

## Abstract

**Background:**

Epilepsy remains a major clinical challenge, suggesting that mechanisms beyond the classical excitation-inhibition imbalance contribute to epilepsy. The Liver kinase B1 (LKB1) signaling pathway coordinates energy homeostasis and cell survival, but its key downstream effectors in epilepsy remain largely unknown.

**Methods:**

We integrated machine learning screening of LKB1 pathway genes using public Gene Expression Omnibus datasets (GSE143272) with *in vivo* and *in vitro* experimental validation. A PTZ-induced status epilepticus rat model, AAV-mediated gene knockdown/overexpression, lentiviral transfection of primary hippocampal neurons, Electroencephalography recording, whole-cell patch-clamp recording, and Western blot analysis were employed to investigate the functional role and molecular mechanisms of candidate genes.

**Results:**

Machine learning identified SIK1 (salt-inducible kinase 1) as the top-ranked epilepsy risk gene within the LKB1 pathway. SIK1 expression was significantly upregulated in the hippocampus of SE rats. AAV-mediated SIK1 knockdown prolonged seizure latency and reduced seizure duration, while SIK1 overexpression exacerbated seizure severity. Mechanistically, through PPI network analysis and mediation analysis, we identified NR4A2 as a downstream effector of SIK1. SIK1 negatively regulated NR4A2 expression: SIK1 knockdown increased NR4A2, whereas SIK1 overexpression decreased NR4A2. Rescue experiments showed that co-overexpression of SIK1 and NR4A2 reversed the pro-epileptic effect of SIK1 alone. Whole-cell patch-clamp recordings demonstrated that SIK1 knockdown or NR4A2 overexpression restored action potential amplitude to near-normal levels in magnesium-free-treated neurons.

**Conclusion:**

SIK1 negatively regulates NR4A2, and restoring NR4A2 expression counteracts SIK1-induced neuronal hyperexcitability. These findings suggest that targeting the SIK1-NR4A2 axis may represent a potential therapeutic strategy for epilepsy.

## Introduction

Epilepsy (EP) is a chronic brain disorder characterized by recurrent epileptic seizures. The traditional view holds that seizures originate from an imbalance between neuronal excitation and inhibition (E-I imbalance) in the brain (Li et al., 2022; Li et al., 2023). However, despite most clinical antiseizure medications (ASMs) aiming to correct this E-I imbalance, a significant proportion of patients respond poorly (Kwan et al., 2010). This suggests that the E-I imbalance theory may not fully explain epileptogenesis. Previous studies have turned its focus to other pathological processes. For instance, evidence indicates that imbalances in brain energy metabolism and the consequent activation of cell death signaling pathways play a key role in seizure occurrence. Notably, correcting energy metabolism deficits or pharmacologically protecting neurons have both been shown to effectively alleviate seizures in pre-clinical models (Li et al., 2025a; Li et al., 2025b; Sada. et al., 2015). These findings collectively suggest that energy imbalance and cell death may represent important new dimensions beyond E-I imbalance for understanding the core pathophysiology of epilepsy.

Among the numerous signaling networks coordinating energy homeostasis and cell survival, the Liver kinase B1 (LKB1) signaling pathway stands out as a critical hub. LKB1 senses cellular energy status by activating AMP-activated kinase (AMPK), thereby regulating metabolic processes and preventing cell damage caused by energy depletion (Herschlag et al., 2009). Studies show that in the context of epilepsy, dysregulation of LKB1 signaling may exacerbate energy metabolic imbalance; for example, in zinc excitotoxicity models, the LKB1-AMPK-Bim cascade is activated, inducing caspase-3-dependent neuronal apoptosis, which is directly linked to cell death mechanisms in seizures (Herschlag et al., 2009). Furthermore, LKB1 dysfunction (e.g., STRADalpha deficiency) leads to hyperactivation of mTORC1 signaling, promoting neuronal heterotopia and epileptiform activity, further highlighting the central role of LKB1 in maintaining energy homeostasis and cell survival (Orlova et al., 2010; Puffenberger et al., 2007). This evidence collectively indicates that the LKB1 signaling pathway is not only a key regulator of energy metabolism but also an important bridge connecting cell death signals in epilepsy pathology, providing a theoretical basis for developing novel anti-epilepsy strategies targeting energy imbalance.

LKB1, as an upstream master kinase, exerts its functions through phosphorylating and regulating a series of downstream substrates (Kim et al., 2020). However, within the specific context of epilepsy, the key mediator molecules of the LKB1 signaling pathway that contribute to epilepsy remain largely unknown. To address this gap, we employed machine learning approaches to systematically screen for LKB1 pathway-related genes that may contribute to epilepsy risk using publicly available patient transcriptome data. Based on this screening, we then proceeded to validate the expression and functional role of the top-ranked candidate gene in an animal model of epilepsy. Furthermore, we sought to explore its potential downstream molecular mechanisms and functional significance in regulating neuronal excitability.

## Methods and materials

### Data acquisition and pre-processing

The datasets were downloaded from the Gene Expression Omnibus (GEO) (www.ncbi.nlm.nih.gov/geo): GSE143272 (Platform: GPL10558), GSE33000 (Platform: GPL4372) and GSE63808 (Platform: GPL6947). The GSE143272 dataset contained 51 blood samples from healthy individuals and 91 blood samples from individuals with EP, which was used as the discovery cohort for machine learning screening. The GSE33000 dataset contained 158 normal brain tissue samples, and the GSE63808 dataset contained 129 epileptic brain tissue samples. These two brain tissue datasets were merged and used as an independent external validation cohort to evaluate the generalizability of the predictive models.

### Constructing a predictive model based on multiple machine-learning (ML) methods

LKB1-related genes (Name: PID_LKB1_PATHWAY) were downloaded from Gene Set Enrichment Analysis (GSEA) (www.gsea-msigdb.org). Based on the LKB1-related genes, the “caret” R package was utilized to establish machine learning models, which included the random forest (RF) model, support vector machine (SVM) model, generalized linear model (GLM), and eXtreme Gradient Boosting (XGB). All models were trained using repeated 5-fold cross-validation to optimize hyperparameters and mitigate the risk of overfitting. The “pROC” R package was used to visualize the area below the ROC curve. The optimal machine learning model was identified, and the top five important variables in LKB1-related genes were considered as the key predictive molecules associated with EP. Next, ROC curve analysis was performed on the training cohort to verify the diagnostic value of the diagnostic model. The predictive performance of the models was further validated using an independent external dataset generated by merging GSE33000 and GSE63808.

### Constructing and validating a nomogram model

Using the “rms” R package, the nomogram models were constructed to evaluate LKB1-related genes. Each predictive molecule had a corresponding score, and the “total score” represented the sum of the scores of all predictive molecules. Calibration curve analysis and decision curve analysis (DCA) were utilized to evaluate the predictive performance of the nomogram.

### SHAP analysis reveals predictive contributions of key variables

To deeply elucidate the contribution mechanisms of key predictive variables in the machine learning model, this study employed SHAP (SHapley Additive exPlanations) method for interpretability analysis of the top five important genes screened from the model. Based on the trained random forest model, we calculated SHAP values for each gene using the fastshap package. Through multiple visualization approaches including feature importance plots, beeswarm plots, and dependence plots, we systematically revealed the specific contribution levels and directional effects of these key genes in classification decisions. Positive SHAP values indicate that gene expression increases the probability of samples being classified into the treatment group, while negative values enhance the classification tendency toward the control group, thereby elucidating the predictive weights and influence mechanisms of key biomarkers at the model level.

### Identify the interrelationships among key predictive variables

The “corrplot” R package was then utilized to conduct correlation analysis on DE-DRMs to explore the correlation between these molecules. Finally, the String website (https://cn.string-db.org/) and GeneMANIA website (http://genemania.org/) was used to conduct protein-protein interaction (PPI) analysis on the key predictive variables.

### Mediation analysis

To identify potential mediators of SIK1, we performed mediation analysis using the mediation R package. Based on the protein-protein interaction network and gene co-expression analysis, candidate genes (including NR4A2, CRTC2, CREB1, and CRTC3) were selected as potential mediators. The mediation model was constructed as follows: the expression level of SIK1 was set as the independent variable (X), the expression level of each candidate gene was set as the mediator (M), and the epilepsy status (control vs. epilepsy) was set as the dependent variable (Y). The mediation effect was estimated using a two-step regression approach. First, a linear regression model was fitted to assess the effect of X on M (path a). Second, a logistic regression model was fitted to assess the effects of X and M on Y (paths b and c'). The average causal mediation effect (ACME) was estimated using bootstrap resampling with 1,000 iterations. A significant ACME (p < 0.05) indicated that the candidate gene significantly mediated the effect of SIK1 on epilepsy risk.

### Constructing the *in vivo* models of status epilepticus (SE)

Male Sprague-Dawley rats weighing 220–250 g (approximately 7–8 weeks old) were used in this study. Rats were randomly assigned to either a normal control group (n = 6) or a group subjected to pentylenetetrazol (PTZ)-induced SE (n = 6). To induce SE, animals in the experimental group received an initial intraperitoneal (i.p.) injection of PTZ at 40 mg/kg, followed by supplemental doses of 20 mg/kg every 10 min until the establishment of SE or until a maximum cumulative dose of 160 mg/kg was reached (Hussenet et al., 1995). Animals that did not develop SE after reaching the maximum cumulative dose were excluded from the analysis, and additional animals were added to maintain n = 6 per group. Animals that died during the procedure were also immediately replaced with additional animals. Seizure latency and duration were recorded. After SE onset, animals were observed for 60 min. If seizures persisted beyond this period, they were terminated with diazepam (10 mg/kg, i. p.) before tissue collection. All procedures were performed by an investigator blinded to the treatment groups. Control rats were administered an equivalent volume of physiological saline. All injections were performed between 10:30 a.m. and 12:30 p.m. Seizure severity was rated according to a standardized scale (Becker. et al., 1992; Racine. et al., 1972) as follows: stage 0, no seizure; stage 1, ear and facial twitching; stage 2, intense myoclonus without an upright posture; stage 3, intense myoclonus with an upright posture and bilateral forelimb clonus; stage 4, clonic-tonic seizures; stage 5, generalized clonic-tonic seizures accompanied by loss of postural control. SE was defined as continuous seizure activity at stage 4 or higher, persisting for a minimum of 30 min, characterized by prolonged convulsive episodes interspersed with postictal depressive periods and the absence of regained consciousness or a quadrupedal stance (Erdogan. et al., 2005). The latent period, representing the interval from the first PTZ injection to the first observed seizure, was determined for each animal. The total duration of seizures at stage 4 or above was also recorded. Subsequently, all rats were deeply anesthetized with isoflurane, and their hippocampi were rapidly dissected for subsequent biochemical analysis.

### Adeno-associated viral (AAV) vector construction and stereotaxic injections

AAV vector constructs tagged with green fluorescent protein (GFP) were procured from Hanheng Co., Ltd. These included an AAV negative control (AAV-NC, titer: 3.12 × 10^13^ VG/mL) an AAV vector encoding a short hairpin RNA (shRNA) targeting SIK1 (shRNA.SIK1, titer: 5.36 × 10^13^ VG/mL). Rats were randomly assigned to two groups: NC (n = 6) and shRNA.SIK1 (n = 6). Prior to viral delivery, the animals were deeply anesthetized with 3% sodium pentobarbital and securely positioned in a stereotaxic instrument using ear bars and a head holder. Each hippocampus was infused with 1 μL of the corresponding AAV solution at an injection rate of 0.2 μL/min, administered via a 5-μL glass syringe (Hamilton 7633–01) coupled with a bevelled needle (Hamilton, 30°, 30 gauge, 0.5″). The stereotaxic coordinates targeted the hippocampus as follows: anterior/posterior (AP), −3.0 mm; lateral/medial (LM), ±2.2°mm; dorsal/ventral (DV), −3.5 mm. The total vector dose per animal was 3.12 × 10^7^ VG for AAV-NC and 5.36 × 10^7^ VG for shRNA.SIK1. Four weeks after AAV injection (to allow sufficient viral expression), all rats in each group were subjected to PTZ-induced seizures via intraperitoneal injection, starting with an initial dose of 40 mg/kg, followed by supplemental doses of 20 mg/kg at 10-min intervals until SE was established. The latency to the first epileptic seizure (seizure latency) following the initial PTZ injection was recorded, along with the Racine scale scores of the seizures in the rats. Animals that died during the procedure were immediately replaced with additional animals to maintain n = 6 per group.

### Electroencephalography (EEG) recordings

The rats were anesthetized with isoflurane to enable noninvasive fixation. Surface electrodes were placed on the forehead (frontal regions), temporal cortex, and both forelimbs. EEG signals were acquired using a Nuochen EEG recording system with the following channel configuration: left frontal (Fp1), right frontal (Fp2), left temporal (T3), and right temporal (T4). Continuous EEG was recorded from each rat for 30 min. Successful model establishment was confirmed by the presence of recurrent interictal epileptiform discharges, generalized multispike waves, and multiple spike-and-slow-wave complexes on EEG.

### Constructing the *in vitro* models of seizures

Primary hippocampal neurons were isolated from the hippocampus of neonatal Sprague-Dawley rats (postnatal day 1). Dissected hippocampal tissues were digested with 0.25% trypsin-EDTA for 15 min at 37 °C, followed by mechanical trituration into single-cell suspensions. Neurons were seeded onto poly-D-lysine-coated coverslips in 24-well plates at a density of 2 × 10^5^ cells per well and maintained in Neurobasal medium supplemented with B27 (2%), GlutaMAX (1%), and penicillin-streptomycin (1%). Cultures were incubated at 37 °C in a humidified atmosphere containing 5% CO_2_, and half of the culture medium was replaced every 3 days.

To induce epileptiform activity, neurons at 15 days *in vitro* (DIV15) were exposed to magnesium-free artificial cerebrospinal fluid (ACSF) for 3 h. The standard ACSF contained (in mM): NaCl 145, KCl 2.5, HEPES 10, CaCl_2_ 2, glucose 10, glycine 0.002, and MgCl_2_ 1. The Mg^2+^-free ACSF had the same composition except that MgCl_2_ was omitted. Neurons cultured in normal ACSF served as controls.

For electrophysiological recordings, glass electrodes (resistance 3–6 MΩ) were pulled from borosilicate capillaries and filled with intracellular solution containing (in mM): KCl 110, NaCl 1, EGTA 2, HEPES 25, Mg-ATP 4, Na_2_-GTP 0.3, and phosphocreatine 10 (pH adjusted to 7.3 with KOH). Extracellular recording solution consisted of (in mM): NaCl 122, KCl 2, HEPES 25, CaCl_2_ 2, MgCl_2_ 4, and glucose 10 (pH adjusted to 7.4 with NaOH) (Zhu et al., 2025; Zhu et al., 2024). Whole-cell patch-clamp recordings were performed at room temperature (25 °C) using a Digidata 1550B system. Neurons were voltage-clamped at −70 mV to record action potentials in current-clamp mode. Six neurons per experimental condition were recorded from at least three independent culture preparations.

### Neuronal transfection

Lentiviral (LV) vectors were purchased from Sangon Biotechnology (Shanghai) Co., Ltd. The following LV constructs were used: LV-negative control (NC, titer: 3.83 × 10^9^ TU/mL), LV-short hairpin RNA targeting SIK1 (shRNA.SIK1, titer: 2.43 × 10^9^ TU/mL), LV-overexpressing SIK1 (OE.SIK1, titer: 3.05 × 10^9^ TU/mL), LV-overexpressing NR4A2 (OE.NR4A2, titer: 4.23× 10^9^ TU/mL), and a combination of OE.SIK1 and OE.NR4A2 (OE.SIK1+OE.NR4A2) for co-transfection experiments. Based on previously reported protocols and preliminary optimization, a multiplicity of infection (MOI) of 5 was used for all transfection experiments (Li et al., 2025a). Primary hippocampal neurons at DIV3 were incubated with the corresponding LV vectors in serum-free Neurobasal medium for 24 h. After incubation, the medium was replaced with fresh Neurobasal medium supplemented with B27. Transfected neurons were cultured until DIV15 for subsequent experiments.

The following experimental groups were established for Western blot validation and electrophysiological recording: (1) NC (negative control); (2) NC + Mg^2+^-free (epilepsy model control); (3) shRNA.SIK1 + Mg^2+^-free; (4) OE.SIK1 + Mg^2+^-free; (5) OE.NR4A2 + Mg^2+^-free; (6) OE.SIK1 + OE.NR4A2 + Mg^2+^-free. Prior to functional assays, Western blot was performed to verify the expression levels of SIK1 and NR4A2 in each transfection group.

### Immunofluorescence (IF) staining

Deparaffinized and rehydrated hippocampal sections underwent antigen retrieval with citrate buffer. After permeabilization with 0.3% Triton X-100 and blocking with 5% normal goat serum, the sections were incubated overnight at 4 °C with the following primary antibodies: anti-salt-inducible kinase 1 (SIK1) antibody (1:100, Omnimabs, OM165322), anti- Nuclear Receptor Subfamily 4, Group A, Member 2 (NR4A2) antibody (1:100, Sangon Biotech, D221063). Subsequently, the sections were incubated with appropriate fluorochrome-conjugated secondary antibodies for 1 h at room temperature. Nuclei were counterstained with DAPI, and the sections were mounted with an anti-fade mounting medium for observation under a confocal laser scanning microscope.

### Western blot analysis

Total protein (Zhang. et al., 2019) of the hippocampus was extracted using a Protein Extraction Kit (Invent Biotechnologies, SD-001/SN-002; SM-005). A BCA assay kit (Beyotime, P0012S) was used to evaluate the concentrations of protein. Protein samples (20 μg protein/lane) were separated by sodium dodecyl-sulfate polyacrylamide gel electrophoresis (SDS-PAGE) and transferred to a polyvinylidene fluoride (PVDF) membrane (Merck Millipore Ltd.) using a wet transfer cell (Bio-Rad). The PVDF membrane was blocked and incubated with the primary antibody at 4 °C overnight. The primary antibodies used were anti-SIK1 antibody 1:1000 (Omnimabs, OM165322), anti-NR4A2 antibody 1:1,000 (Boster, PB0332) and anti-alpha Tubulin antibody 1:10000 (Abcam, ab52866). Subsequently, the PVDF membrane was incubated with IRDye 800CW-conjugated goat anti-rabbit immunoglobulin IgG (product#C70301-05, 1:2000, CST) for 1 h at room temperature. OdysseyInfrared Imaging System (LI-COR Biosciences) was used to obtain experimental results. Protein levels were determined by normalization to GAPDH/Tubulin loading controls. Each experiment was repeated three times.

### Statistical analyses

For *in vivo* studies, n represents the number of independent animals per group. For *in vitro* whole-cell patch-clamp recordings, n represents the number of neurons recorded from at least three independent culture preparations. For Western blot and other biochemical assays, n represents the number of independent biological replicates.

Statistical analyses were performed using SPSS 25.0 software. Data distribution was assessed for normality using the Shapiro-Wilk test, and homogeneity of variance was evaluated using Levene’s test. For comparisons between two groups, the independent sample t-test was applied. For comparisons among multiple groups, one-way ANOVA was performed followed by Tukey’s *post hoc* test for multiple comparisons. All data are presented as mean ± standard deviation. A p-value <0.05 was considered statistically significant.

## Results

### Identification of key genes for epilepsy risk based on machine learning

The residuals of the four machine learning models constructed based on LKB1-related genes are shown in Figure 1A, with the SVM model identified as the optimal ML model (AUC = 0.884, Figure 1B). The top 15 most important genes from the four ML models are displayed in Figure 1C. External validation using the merged GSE33000 and GSE63808 dataset showed that RF achieved an AUC of 0.864 (Figure 1D), outperforming SVM (AUC = 0.780) (Figure 1E), suggesting that RF may have better generalizability, although this should be interpreted with caution given the differences in brain region and tissue collection between the two datasets. Based on the RF model, the top five ranked genes were selected as key epilepsy risk genes (PRKAB1, CTSD, SMARCD3, MLST8, SIK1) to construct a nomogram model (Figure 1F). We observed that the risk of epilepsy increased as the expression levels of PRKAB1 and CTSD decreased, whereas the risk increased with higher expression levels of SMARCD3, MLST8, and SIK1.

**FIGURE 1:**
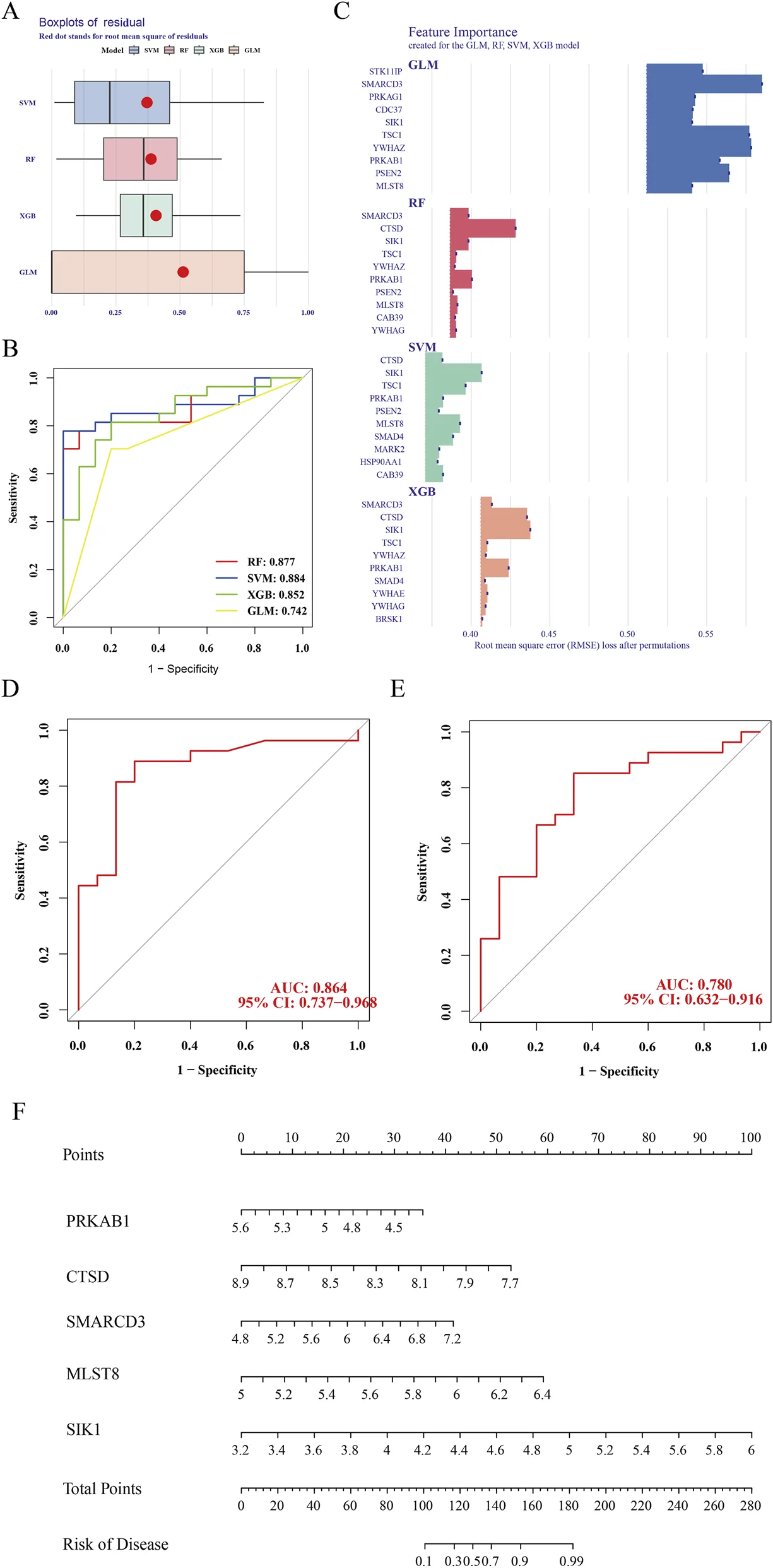
Identification of LKB1 pathway genes for epilepsy risk based on machine learning. **(A)** Residual plots of the four machine learning models. **(B)** ROC curves of the models (AUC.RF = 0.877; AUC. SVM = 0.884; AUC. XGB = 0.852; AUC. GLM = 0.742). **(C)** Ranking of LKB1 pathway gene importance in each model. **(D)** External validation of the RF model using the merged GSE33000 (*postmortem* prefrontal cortex) and GSE63808 (surgically resected hippocampus) dataset (AUC = 0.864). **(E)** External validation of the SVM model using the same merged dataset (AUC = 0.780). **(F)** Nomogram for predicting epilepsy risk based on the 5-gene RF model (PRKAB1, CTSD, SMARCD3, MLST8, SIK1).

### SHAP analysis of the importance of key epilepsy risk genes

Based on SHAP analysis, the importance of the five key genes (SIK1, MLST8, SMARCD3, PRKAB1, CTSD) is shown in Figure 2A, with SIK1 having the highest SHAP value. The SHAP dependence plots for the five genes are displayed in Figures 2B–F. The SHAP values for SIK1 and MLST8 increased with higher gene expression levels (Figures 2B,D), while the relationships between SHAP values and gene expression for SMARCD3 and PRKAB1 were not purely linear (Figures 2C,E). The SHAP value for CTSD decreased with increasing gene expression (Figure 2F). Given that SIK1 exhibited the highest SHAP value among the five candidate genes, we focused on SIK1 for further mechanistic exploration.

**FIGURE 2:**
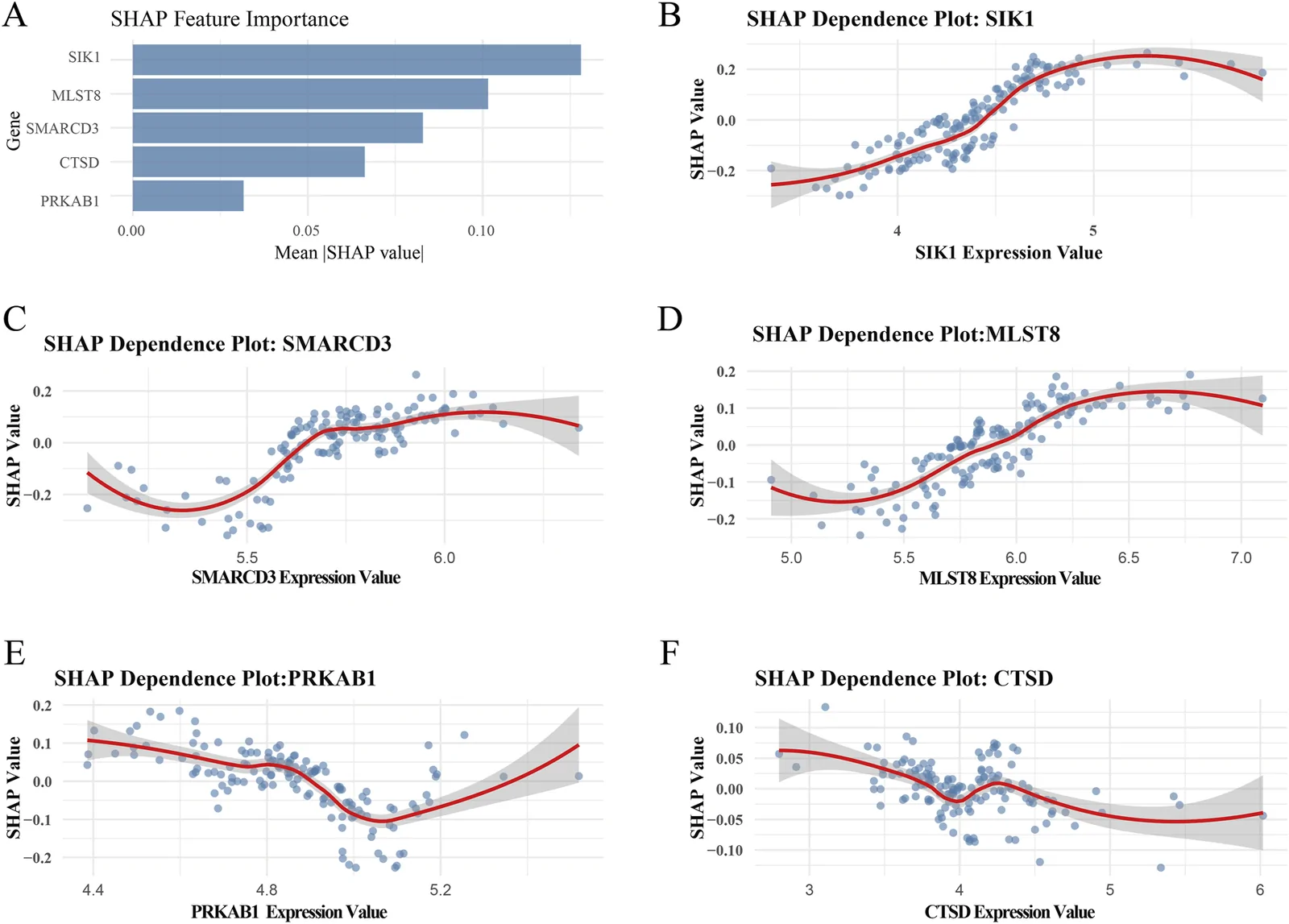
SHAP analysis of key epilepsy risk genes. **(A)** SHAP values of the five key genes (SIK1, MLST8, SMARCD3, PRKAB1, CTSD). **(B–F)** SHAP dependence plots for SIK1 **(B)**, SMARCD3 **(C)**, MLST8 **(D)**, PRKAB1 **(E)**, and CTSD **(F)**.

### Mediation analysis identifies NR4A2 as a potential mediator of SIK1

To explore potential interacting partners of SIK1, we constructed a gene interaction network using GeneMANIA and a PPI network using STRING. As shown in Figures 3A,B, SIK1 was predicted to interact with several candidate genes/proteins. We then performed mediation analysis to evaluate whether these candidate genes mediated the effect of SIK1 on epilepsy risk. Remarkably, among all candidates, only NR4A2 showed a significant mediating effect (ACME p < 0.001, Figure 3C; Supplementary Tables S1,S2), suggesting that NR4A2 may serve as a key mediator of SIK1 in epilepsy. Furthermore, analysis of the GEO dataset confirmed the expression of both SIK1 and NR4A2 in control and epilepsy samples (Figure 3D).

**FIGURE 3:**
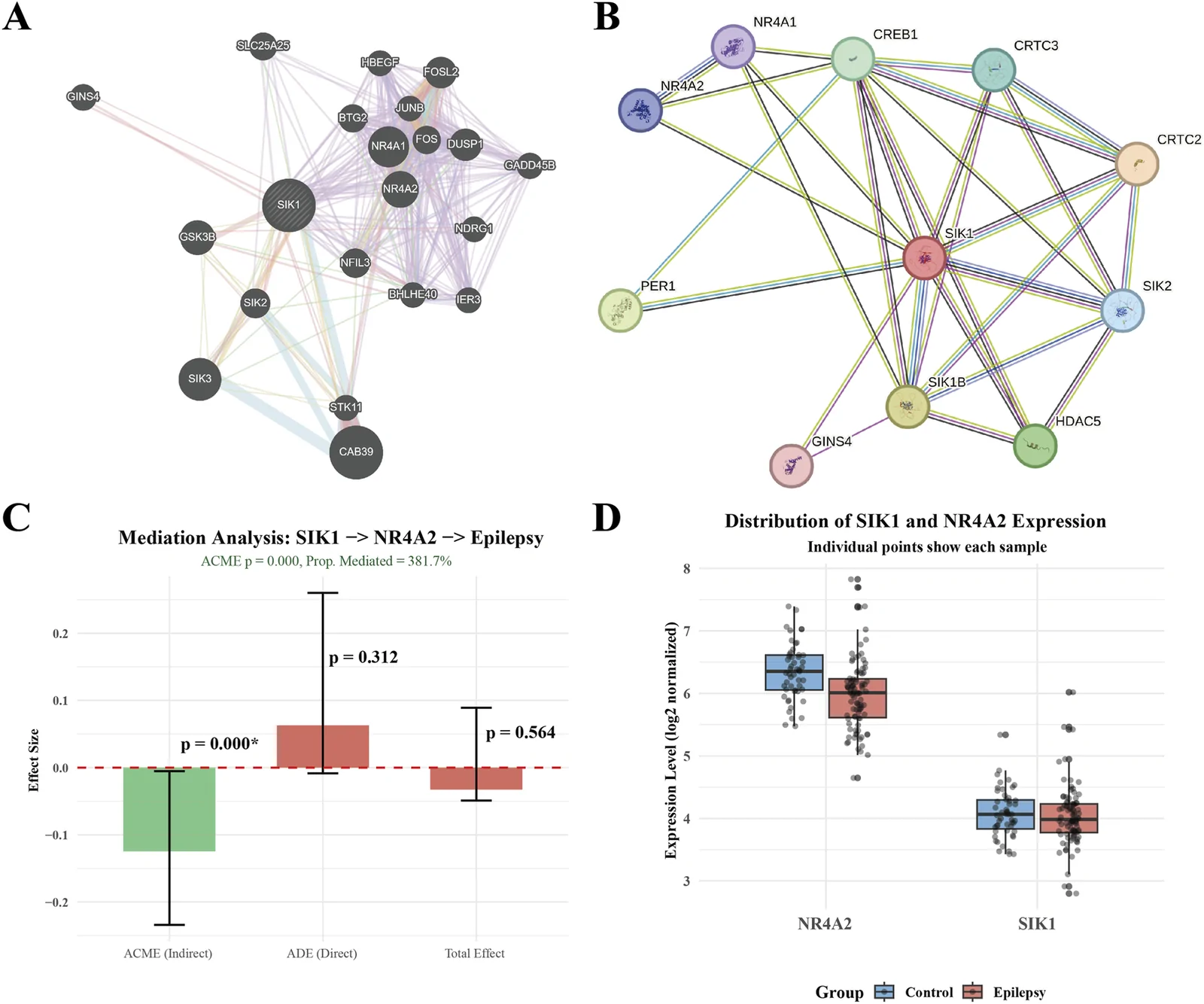
Identification of NR4A2 as a potential mediator of SIK1. **(A)** Gene interaction network of SIK1 constructed using GeneMANIA. **(B)** Protein-protein interaction (PPI) network of SIK1 from the STRING database. **(C)** Mediation analysis showing that NR4A2 significantly mediates the effect of SIK1 on epilepsy risk (ACME p < 0.001). **(D)** Expression of SIK1 and NR4A2 in control and epilepsy samples from the GSE143272 dataset (51 healthy controls and 91 epilepsy patients, peripheral blood). Statistical comparisons were performed using the independent sample t-test.

### SIK1 plays a key role in seizures

Immunofluorescence analysis of rat hippocampi revealed that SIK1 and NR4A2 are all expressed in the rat hippocampus (Figure 4A). EEG recordings from the two rat groups are shown in Figure 4B. No significant epileptiform discharges were observed in the control group rats, whereas the SE group rats exhibited significant spikes, spike-wave complexes, and other epileptiform discharges. Additionally, the duration of seizures in the SE group rats exceeded 30 min. Compared to the Ctrl group, the SE group showed significantly increased expression of SIK1 (n = 6; vs. Ctrl, p < 0.01, Figure 4C), and significantly decreased expression of NR4A2 and CTSD (n = 6; vs. Ctrl, p < 0.01, Figure 4C). Subsequently, we performed stereotaxic injections into the rat hippocampus and challenged the rats with PTZ. Western blot results showed that SIK1 expression was decreased in shRNA.SIK1 group compared to the NC group (n = 6; vs. NC, p < 0.01, Figure 4D), while the expression of NR4A2 was increased in the shRNA.SIK1 group compared to the NC group (n = 6; vs. NC, p < 0.01, Figure 4D). EEG recordings from both groups showed epileptiform discharges (Figure 4E). In the shRNA.SIK1 group, seizure latency was significantly prolonged and seizure duration was significantly shortened compared to the NC group (n = 6; vs. NC, p < 0.01, Figure 4F).

**FIGURE 4:**
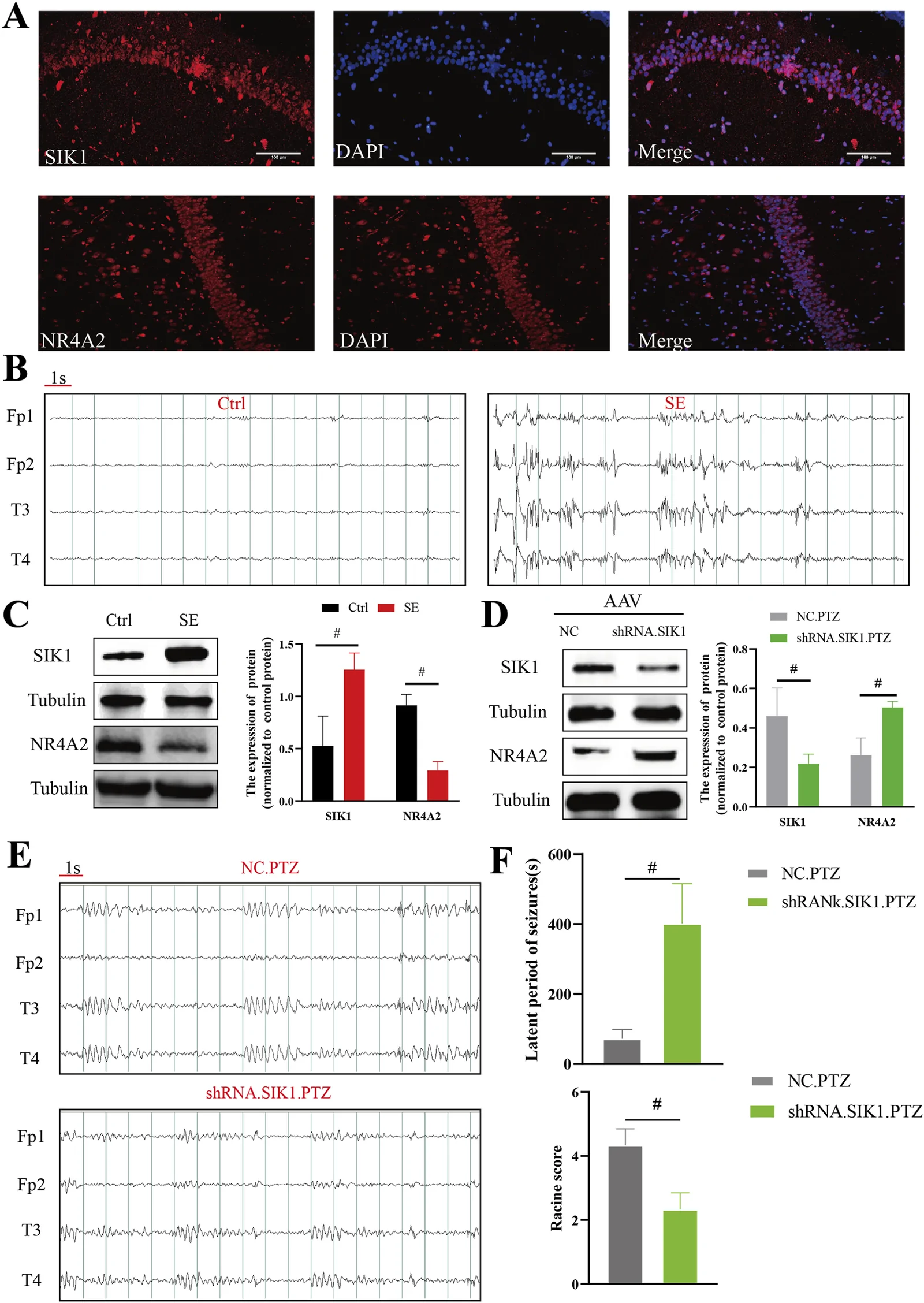
SIK1 Plays a Key Role in Seizures **(A)** Immunofluorescence staining of SIK1 and NR4A2 in rat hippocampus (×400, scale bar: 200 μm). **(B)** EEG recordings showing no epileptiform discharges in the control group but significant discharges in the SE group. **(C)** Western blot showing increased SIK1 expression and decreased NR4A2 expression in the SE group compared to control (n = 6; p < 0.01). **(D)** Western blot validation of SIK1 knockdown efficiency and NR4A2 expression changes (n = 6; p < 0.01). **(E)** EEG recordings from NC and shRNA.SIK1 groups after PTZ challenge. **(F)** shRNA.SIK1 significantly prolonged seizure latency and shortened seizure duration compared to the NC group (n = 6; #, p < 0.01).

### SIK1 exacerbates seizures by suppressing NR4A2

To validate the regulatory relationship between SIK1 and NR4A2, we performed Western blot analysis on primary hippocampal neurons transfected with different LV constructs. As shown in Figure 5A, knockdown of SIK1 (shRNA.SIK1) significantly increased NR4A2 expression, whereas overexpression of SIK1 (OE.SIK1) significantly decreased NR4A2 expression, confirming a negative regulatory relationship between SIK1 and NR4A2 (n = 6; p < 0.01, Figure 5A). In the rescue experiment, OE.NR4A2 alone increased NR4A2 expression without affecting SIK1 levels. Co-transfection of OE.SIK1 and OE.NR4A2 successfully overexpressed both genes, serving as a rescue model (n = 6; p < 0.01, Figure 5B). We next performed whole-cell patch-clamp recordings on primary hippocampal neurons subjected to Mg^2+^-free treatment to induce epileptiform activity. Representative action potential traces and quantitative analyses of firing frequency and amplitude are shown in Figure 5C. As expected, magnesium-free treatment significantly increased both the frequency and amplitude of action potentials in NC neurons compared to the control condition (n = 6; NC + Mg^2+^-free vs. NC, p < 0.01), confirming the successful establishment of the *in vitro* epilepsy model. Knockdown of SIK1 (shRNA.SIK1 + Mg^2+^-free) significantly reduced the firing amplitude to levels comparable to the NC control; however, the firing frequency remained elevated. In contrast, overexpression of SIK1 (OE.SIK1 + Mg^2+^-free) further increased both firing frequency and amplitude, resulting in the most severe neuronal hyperexcitability among all groups. Overexpression of NR4A2 alone (OE.NR4A2 + Mg^2+^-free) restored firing amplitude to near-normal levels, phenocopying the effect of SIK1 knockdown, while firing frequency remained elevated. Strikingly, co-overexpression of SIK1 and NR4A2 (OE.SIK1 + OE.NR4A2 + Mg^2+^-free) reversed the exacerbating effect of SIK1 overexpression alone, normalizing the firing amplitude to levels similar to the OE.NR4A2 group, although the firing frequency was still increased (Figure 5C).

**FIGURE 5:**
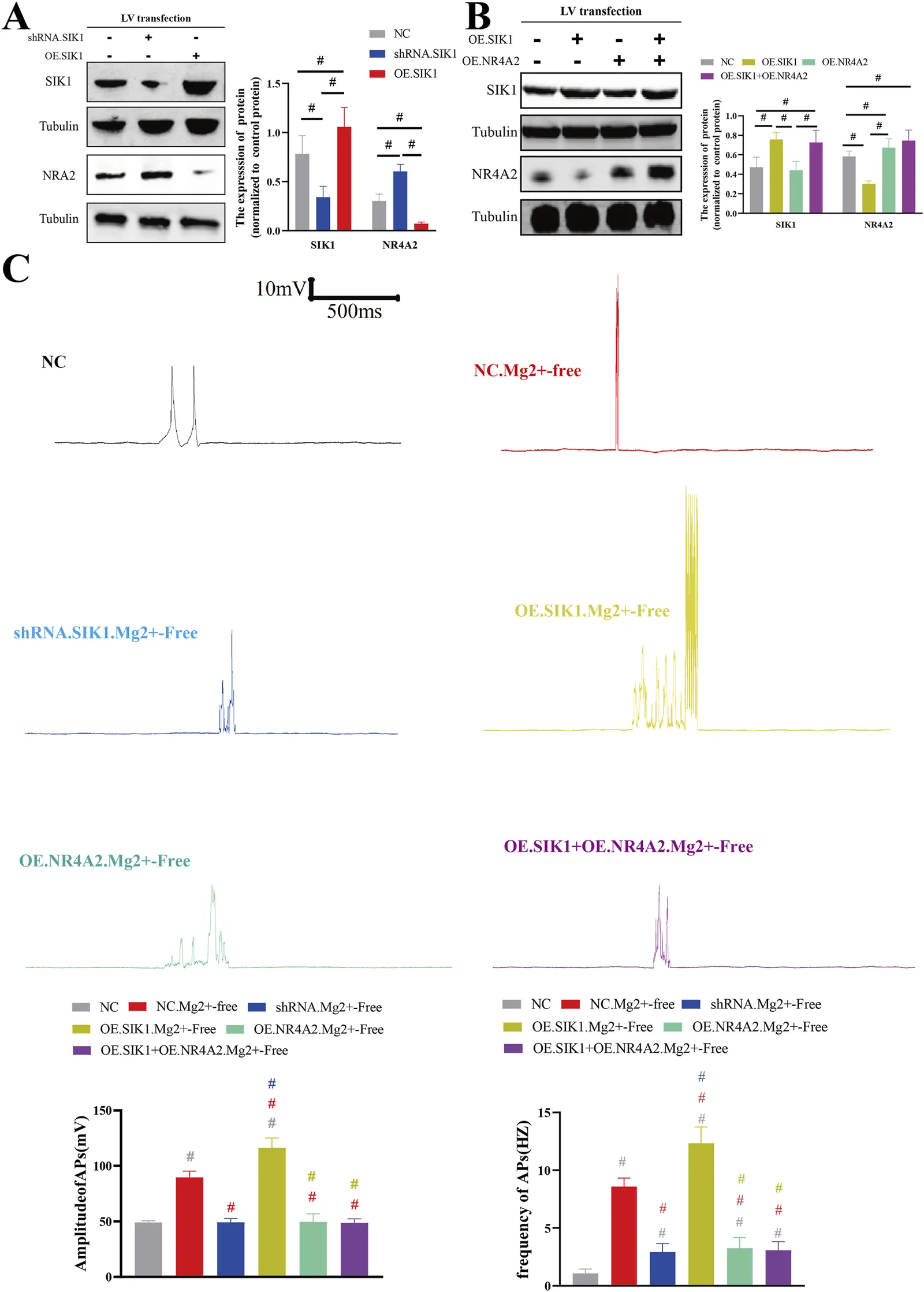
SIK1 exacerbates seizures by suppressing NR4A2. **(A)** Western blot showing that shRNA.SIK1 increases NR4A2 expression, while OE.SIK1 decreases NR4A2 expression (n = 6; #, p < 0.01). **(B)** Rescue experiment: OE.NR4A2 alone increases NR4A2 without affecting SIK1; co-transfection of OE.SIK1 and OE.NR4A2 overexpresses both genes (n = 6; #, p < 0.01). **(C)** Representative action potential traces and quantitative analyses of firing frequency and amplitude from whole-cell patch-clamp recordings. Mg^2+^-free treatment increases both frequency and amplitude in NC neurons. shRNA.SIK1 or OE.NR4A2 alone restores amplitude but not frequency; OE.SIK1 exacerbates both; co-overexpression of SIK1 and NR4A2 reverses the amplitude abnormality (n = 6; #, p < 0.01).

## Discussion

Despite decades of research, the molecular mechanisms underlying epilepsy remain incompletely understood, particularly regarding the specific mediators of the LKB1 signaling pathway that contribute to seizure generation. In this study, we employed an integrated approach combining machine learning screening of LKB1 pathway genes with *in vivo* and *in vitro* experimental validation to investigate the functional roles of candidate genes in epileptogenesis.

Machine learning screening of LKB1 pathway genes identified SIK1 (salt-inducible kinase 1) as a top-ranked candidate associated with epilepsy risk. SIK1 is an AMPK-related kinase that responds to changes in cellular energy status and cAMP-dependent signaling, playing roles in neuronal excitability and stress responses (Hou et al., 2024; Hu et al., 2015; Yang et al., 2025). SHAP interpretability analysis further revealed a positive correlation between SIK1 expression levels and its SHAP value, indicating that higher SIK1 expression contributed more strongly to epilepsy classification decisions. To validate this computational prediction, we examined SIK1 expression in a PTZ-induced status epilepticus (SE) rat model. Consistent with the machine learning prediction, SIK1 expression was significantly upregulated in the hippocampus of SE rats compared to controls. To functionally assess the role of SIK1 in seizures, we performed AAV-mediated knockdown of SIK1 in the rat hippocampus. Knockdown of SIK1 significantly prolonged seizure latency and reduced seizure duration, while overexpression of SIK1 had the opposite effect, further exacerbating seizure severity. Consistently, *in vitro* lentiviral transfection of primary hippocampal neurons followed by magnesium-free treatment showed that SIK1 knockdown significantly reduced action potential amplitude, whereas SIK1 overexpression further increased both firing frequency and amplitude. These results establish SIK1 as a critical functional driver of seizures. Given these findings, we next sought to explore the molecular mechanisms downstream of SIK1.

To identify downstream molecular targets of SIK1, we performed gene interaction network and PPI analysis and identified NR4A2 as a potential interacting partner of SIK1. Mediation analysis further revealed that NR4A2 significantly mediated the effect of SIK1 on epilepsy risk, suggesting that NR4A2 may serve as a key downstream effector of SIK1 in epilepsy. To validate this regulatory relationship, we performed lentiviral transfection of primary hippocampal neurons. Western blot analysis confirmed that SIK1 negatively regulates NR4A2 expression: knockdown of SIK1 significantly increased NR4A2 expression, whereas overexpression of SIK1 significantly decreased NR4A2 expression. In the rescue experiment, co-transfection of SIK1 and NR4A2 overexpression constructs successfully overexpressed both genes, providing a valid model for functional rescue. We next examined the functional significance of the SIK1-NR4A2 axis in neuronal excitability using whole-cell patch-clamp recordings in magnesium-free-treated neurons. SIK1 knockdown or NR4A2 overexpression alone restored action potential amplitude to near-normal levels, phenocopying the protective effect, while SIK1 overexpression further exacerbated both frequency and amplitude abnormalities. Strikingly, co-overexpression of SIK1 and NR4A2 reversed the exacerbating effect of SIK1 alone, normalizing the firing amplitude. Notably, while amplitude was effectively restored by these interventions, firing frequency remained elevated, suggesting that amplitude and frequency may be regulated by distinct molecular mechanisms. However, it should be noted that the Mg^2+^-free neuronal model primarily reflects acute neuronal hyperexcitability and may not fully capture the chronic adaptive changes occurring in epileptic networks.

It should be noted that in the GSE143272 peripheral blood dataset, SIK1 expression did not show a significant increase in the epilepsy group. This may reflect the difference between peripheral blood transcriptional signatures and brain tissue molecular alterations. In contrast, SIK1 protein expression was significantly upregulated in the hippocampus of PTZ-treated rats, suggesting that SIK1 upregulation may be more pronounced in brain tissue than in peripheral blood. This discrepancy highlights the importance of tissue-specific validation and warrants further investigation.

These results demonstrate that NR4A2 is a critical mediator through which SIK1 promotes seizure-like neuronal firing. NR4A2 (also known as Nurr1) is a nuclear receptor transcription factor involved in neuronal development and function. Recent clinical genetic studies have identified *de novo* missense and loss-of-function variants in NR4A2 in patients with neurodevelopmental disorders and epilepsy, with six out of nine unrelated patients presenting with epilepsy (Singh et al., 2020). Additionally, a systematic review has proposed NR4A2 as a first-tier target gene for developmental and epileptic encephalopathy (Gabaldon-Albero et al., 2024), and another study demonstrated that NR4A2 upregulation protects against post-traumatic epilepsy (Xiao et al., 2025). These clinical and experimental findings strongly align with our observation that the expression of NR4A2 is decreased in the epileptic brain and that restoring the expression of NR4A2 counteracts SIK1-induced neuronal hyperexcitability. Together, these data establish the SIK1-NR4A2 axis as a key pathway in epilepsy, with SIK1 acting as a negative regulator of NR4A2.

The findings of this study have clear potential clinical implications. Currently, about one-third of epilepsy patients are resistant to traditional ASMs targeting ion channels or neurotransmitters (Li et al., 2022; Li et al., 2023; Macdonald. and Kelly, 1995; Pisani. et al., 1999), underscoring the need for novel therapeutic strategies. Our work suggests that targeting the SIK1-NR4A2 axis may offer a promising direction for developing novel anti-epilepsy therapies. However, given that our *in vivo* experiments were performed exclusively in an acute PTZ-induced seizure model, the translational relevance of these findings to chronic epilepsy or pharmacoresistant epilepsy remains to be established. Certainly, this study has limitations. First, we used a PTZ-induced acute seizure model. While this model is widely accepted for studying seizure mechanisms, it primarily reflects acute seizure susceptibility rather than chronic spontaneous epilepsy (Akula et al., 2009; Meenu et al., 2019). We only validated the functional effects of SIK1 in the PTZ model and did not examine other chemoconvulsant models such as KA or pilocarpine. Whether the SIK1-NR4A2 axis plays a similar role in other models warrants further investigation. Second, although we established that SIK1 negatively regulates NR4A2, the precise molecular mechanism by which SIK1 suppresses NR4A2 expression remains to be elucidated. Whether SIK1 directly phosphorylates NR4A2 or acts through intermediate transcription factors requires further investigation. Third, we did not examine SIK1 mRNA levels or whether endogenous SIK1 gene mutations occur in the PTZ model. These are valuable directions for future research. Fourth, the external validation cohort was generated by merging GSE33000 (*postmortem* prefrontal cortex samples) and GSE63808 (surgically resected hippocampal tissue). These datasets differ in brain region, tissue collection method, and platform, which may partly contribute to the observed external validation performance. Therefore, the external validation should be considered preliminary, and further validation using more comparable case-control datasets is warranted. Additionally, the differential regulation of action potential amplitude and frequency observed in our patch-clamp recordings remains mechanistically unclear and requires future investigation.

## Conclusion

In conclusion, by integrating machine learning screening with *in vivo* and *in vitro* experimental validation, this study identifies the SIK1-NR4A2 axis as a previously unrecognized regulator in epilepsy. We demonstrate that SIK1 is significantly upregulated in epilepsy and negatively regulates NR4A2, a nuclear receptor transcription factor implicated in neurodevelopmental disorders and epilepsy by recent clinical genetic studies. Mechanistically, SIK1 knockdown or NR4A2 overexpression protects against seizure-like neuronal firing, while restoring NR4A2 expression reverses the pro-epileptic effect of SIK1. These findings suggest that the SIK1-NR4A2 axis may represent a critical pathway in epilepsy and provide a potential rationale for targeting this axis as a novel therapeutic strategy, though further validation in chronic models and mechanistic studies is warranted.

## Data Availability

The original contributions presented in the study are included in the article/Supplementary Material, further inquiries can be directed to the corresponding author.

## References

[B1] Akula K. K. Dhir A. Kulkarni S. K. (2009). Effect of various antiepileptic drugs in a pentylenetetrazol-induced seizure model in mice. Methods Find. Exp. Clin. Pharmacol. 31, 423–432. 10.1358/mf.2009.31.7.1393610 19907717

[B2] Becker A. Gercksch G. Ruthrich H.-L. Pohle W. Marx B. Matthies H. (1992). Kindling and its consequences on learning in rats. Behav. Neural Biol. 57, 37–43. 10.1016/0163-1047(92)90735-m 1567332

[B3] Erdogan F. Golgeli A. Kucuk A. Arman F. Karaman Y. Ersoy A. (2005). Effects of pentylenetetrazole-induced status epilepticus on behavior, emotional memory and learning in immature rats. Epilepsy Behav. 6, 537–542. 10.1016/j.yebeh.2005.03.002 15907747

[B4] Gabaldon-Albero A. Mayo S. Martinez F. (2024). NR4A2 as a novel target gene for developmental and epileptic encephalopathy: a systematic review of related disorders and therapeutic strategies. Int. J. Mol. Sci. 25, 5198. 10.3390/ijms25105198 38791237 PMC11120677

[B5] Herschlag D. Zeqiraj E. Filippi B. M. Goldie S. Navratilova I. Boudeau J. (2009). ATP and MO25α regulate the conformational state of the STRADα pseudokinase and activation of the LKB1 tumour suppressor. PLoS Biol. 7, e1000126. 10.1371/journal.pbio.1000126 19513107 PMC2686265

[B6] Hou Q. Hu W. Peterson L. Gilbert J. Liu R. Man H.-Y. (2024). SIK1 downregulates synaptic AMPA receptors and contributes to cognitive defects in Alzheimer’s disease. Mol. Neurobiol. 61, 10365–10380. 10.1007/s12035-024-04177-6 38727976

[B7] Hu Z. Hu J. Shen W.-J. Kraemer F. B. Azhar S. (2015). A novel role of salt-inducible kinase 1 (SIK1) in the post-translational regulation of scavenger receptor class B type 1 activity. Biochemistry 54, 6917–6930. 10.1021/acs.biochem.5b00147 26567857

[B8] Hussenet F. Boyet S. Nehlig A. (1995). Long-term metabolic effects of pentylenetetrazol-induced status epilepticus in the immature rat. Neuroscience 67, 455–461. 10.1016/0306-4522(95)00062-n 7675178

[B9] Kim Y.-H. Eom J.-W. Koh J.-Y. (2020). Mechanism of zinc excitotoxicity: a focus on AMPK. Front. Neurosci. 14, 577958. 10.3389/fnins.2020.577958 33041769 PMC7522467

[B10] Kwan P. Arzimanoglou A. Berg A. T. Brodie M. J. Allen Hauser W. Mathern G. (2010). Definition of drug resistant epilepsy: consensus proposal by the ad hoc task force of the ILAE commission on therapeutic strategies. Epilepsia 51, 1069–1077. 10.1111/j.1528-1167.2009.02397.x 19889013

[B11] Li S. Huang H. Wei X. Ye L. Ma M. Ling M. (2022). The recycling of AMPA receptors/GABAa receptors is related to neuronal excitation/inhibition imbalance and may be regulated by KIF5A. Ann. Transl. Med. 10, 1103. 10.21037/atm-22-4337 36388788 PMC9652568

[B12] Li S. Wei X. Huang H. Ye L. Ma M. Sun L. (2023). Neuroplastin exerts antiepileptic effects through binding to the α1 subunit of GABA type A receptors to inhibit the internalization of the receptors. J. Transl. Med. 21, 707. 10.1186/s12967-023-04596-4 37814294 PMC10563248

[B13] Li S. He J. Kuang H. Wang X. Zhou M. Li D. (2025a). Rab11a-dependent recycling of Glut3 inhibits seizure-induced neuronal disulfidptosis by alleviating glucose deficiency. Cell Biosci. 15, 69. 10.1186/s13578-025-01396-9 40437641 PMC12121293

[B14] Li S. Sun L. Huang H. Wei X. Lu Y. Qian K. (2025b). Identifying disulfidptosis-related biomarkers in epilepsy based on integrated bioinformatics and experimental analyses. Neurobiol. Dis. 205, 106789. 10.1016/j.nbd.2025.106789 39805370

[B15] Macdonald R. L. Kelly K. M. (1995). Antiepileptic drug mechanisms of action. Epilepsia 36, S2–S12. 10.1111/j.1528-1157.1995.tb05996.x 8784210

[B16] Meenu M. Reeta K. H. Dinda A. K. Kottarath S. K. Gupta Y. K. (2019). Evaluation of sodium valproate loaded nanoparticles in acute and chronic pentylenetetrazole induced seizure models. Epilepsy Res. 158, 106219. 10.1016/j.eplepsyres.2019.106219 31726286

[B17] Orlova K. A. Parker W. E. Heuer G. G. Tsai V. Yoon J. Baybis M. (2010). STRADα deficiency results in aberrant mTORC1 signaling during corticogenesis in humans and mice. J. Clin. Investigation 120, 1591–1602. 10.1172/JCI41592 20424326 PMC2860905

[B18] Pisani F. Oteri G. Russo M. F. Perri R. D. Perucca E. Richens A. (1999). The efficacy of valproate-lamotrigine comedication in refractory complex partial seizures: evidence for a pharmacodynamic interaction. Epilepsia 40, 1141–1146. 10.1111/j.1528-1157.1999.tb00832.x 10448829

[B19] Puffenberger E. G. Strauss K. A. Ramsey K. E. Craig D. W. Stephan D. A. Robinson D. L. (2007). Polyhydramnios, megalencephaly and symptomatic epilepsy caused by a homozygous 7-kilobase deletion in LYK5. Brain 130, 1929–1941. 10.1093/brain/awm100 17522105

[B20] Racine R. Okujava V. Chipashvili S. (1972). Modification of seizure activity by electrical stimulation. 3. Mechanisms. Electroencephalogr. Clin. Neurophysiology 32, 295–299. 10.1016/0013-4694(72)90178-2 4110398

[B21] Sada N. Lee S. Katsu T. Otsuki T. Inoue T. (2015). Targeting LDH enzymes with a stiripentol analog to treat epilepsy. Science 347, 1362–1367. 10.1126/science.aaa1299 25792327

[B22] Singh S. Gupta A. Zech M. Sigafoos A. N. Clark K. J. Dincer Y. (2020). *De novo* variants of NR4A2 are associated with neurodevelopmental disorder and epilepsy. Genet. Med. 22, 1413–1417. 10.1038/s41436-020-0815-4 32366965 PMC7394879

[B23] Xiao M. Wang X. Xiao E. Ming Q. (2025). Overexpression of FTO alleviates traumatic brain injury induced posttraumatic epilepsy by upregulating NR4A2 expression via m6A demethylation. Funct. and Integr. Genomics 25, 17. 10.1007/s10142-024-01522-9 39825968

[B24] Yang Y. Cai X. Zhou M. Chen Y. Pi J. Zhao M. (2025). Association of left ventricular function with cerebral small vessel disease in a community-based population. CNS Neurosci. and Ther. 31, e70226. 10.1111/cns.70226 39976242 PMC11840704

[B25] Zhang S.-H. Liu D.-X. Wang L. Li Y.-H. Wang Y.-H. Zhang H. (2019). A CASPR1-ATP1B3 protein interaction modulates plasma membrane localization of Na(+)/K(+)-ATPase in brain microvascular endothelial cells. J. Biol. Chem. 294, 6375–6386. 10.1074/jbc.RA118.006263 30792309 PMC6484117

[B26] Zhu Q. Long Q. Wei C. Chen J. Nong L. Qin J. (2024). Lactate dehydrogenase-1 may play a key role in the brain energy disturbance caused by cryptococcal meningitis. J. Microbiol. Immunol. Infect. 57, 887–895. 10.1016/j.jmii.2024.08.009 39214781

[B27] Zhu Q. He H. Long Q. Wei C. Chen J. Nong L. (2025). Lactate-Dehydrogenase-5 may play a key role in the disturbance of brain energy caused by tuberculous meningitis. J. Integr. Neurosci. 24, 26741. 10.31083/JIN26741 40302261

